# Complete Genome Sequence of *Pseudomonas aeruginosa* Phage AAT-1

**DOI:** 10.1128/genomeA.00165-16

**Published:** 2016-08-25

**Authors:** Andrés Andrade-Domínguez, Roberto Kolter

**Affiliations:** Department of Microbiology and Immunobiology, Harvard Medical School, Boston, Massachusetts, USA

## Abstract

Aspects of the interaction between phages and animals are of interest and importance for medical applications. Here, we report the genome sequence of the lytic *Pseudomonas* phage AAT-1, isolated from mammalian serum. AAT-1 is a double-stranded DNA phage, with a genome of 57,599 bp, containing 76 predicted open reading frames.

## GENOME ANNOUNCEMENT

It has been shown that vertebrates are widely exposed to phages, which penetrate them quite freely (1, 2). Recently, it was shown that phages can adhere to mucus and provide a non-host-derived antimicrobial defense on the mucosal surfaces of animals (3).

Here, we report the genome sequence of the lytic phage AAT-1, which was isolated from fetal bovine serum using *Pseudomonas aeruginosa* PA14 as the host strain. This phage was able to infect 17 of 23 *P. aeruginosa* clinical and environmental isolates.

The phage genome was sequenced using the Illumina MiSeq system at the MGH sequencing DNA core facility (Cambridge, MA, USA). The 100-bp reads were *de novo* assembled using Velvet (4). The coverage was on the order of 1,000× and two contigs were obtained. Primers were designed from the ends of contigs with an outward orientation and used in PCR, with the genomic DNA of phages used as templates. The sequences of PCR products were determined by Sanger sequencing.

Annotation of open reading frames was performed with RAST (5) and PHAST (6). Sequence similarity searches were performed with the translation of each predicted coding sequence against the NCBI protein database, using BLASTp (7), in order to assign putative protein functions. tRNAscan-SE (8) was used for tRNA annotation, but no putative genes coding for tRNAs were found in phage AAT-1.

Phage AAT-1 has a genome of 57,599 bp, with a coding percentage of 92.61% and a G+C content of 65.84%. In a dot plot alignment, the AAT-1 genome showed a completely colinear similarity and 91% overall nucleotide identity to *Pseudomonas* phage PaMx28 (GenBank accession no. JQ067089.2), isolated from sewage in central Mexico. Because *Pseudomonas* is a bacterium that lives in different environments and is an opportunistic pathogen of mammals, it is not surprising that phages AAT-1 and PaMx28 are closely related.

We predicted 76 unique coding sequences, of which 34 were assigned a predicted function and 42 are hypothetical. We identified genes for DNA replication, including a DNA ligase (AAT1_02003), a putative helicase (AAT1_02051), a DNA polymerase (AAT1_02053), a putative primase/polymerase (AAT1_02071), and the small terminase subunit (AAT1_02072). A predicted HNH-type intron broke the large terminase gene into two fragments (AAT1_02073 and AAT1_02076), and 15 genes for head and tail morphogenesis were identified. Furthermore, we identified genes that encode proteins for DNA and nucleotide metabolism, such as a GTP cyclohydrolase II (AAT1_02009), a dCMP deaminase (AAT1_02044), a thymidylate synthase (AAT1_02046), a nucleotide pyrophosphohydrolase (AAT1_02047), a nucleotide triphosphate hydrolase (AAT1_02063), and a ribonucleotide reductase (AAT1_02001). The genes for host-cell lysis encode for an endolysin (AAT1_02033), an i-spanin (AAT1-02034), and an o-spanin (AAT1-02035).

### Accession number(s).

The complete genome of *P. aeruginosa* phage AAT-1 was deposited in GenBank under the accession number KU204984. The version described in this paper is version KU204984.2.
